# The Effect of Cerebral Small Vessel Disease on the Subtypes of Mild Cognitive Impairment

**DOI:** 10.3389/fpsyt.2021.685965

**Published:** 2021-07-16

**Authors:** Xudong Li, Miaoxin Shen, Yi Jin, Shuhong Jia, Zhi Zhou, Ziling Han, Xiangfei Zhang, Xiaopeng Tong, Jinsong Jiao

**Affiliations:** ^1^Department of Cognitive Disorder, Beijing Tiantan Hospital, Capital Medical University, Beijing, China; ^2^China National Clinical Research Center for Neurological Diseases, Beijing, China; ^3^Medical School, Xizang Minzu University, Xianyang, China; ^4^Department of Neurology, China-Japan Friendship Hospital, Beijing, China

**Keywords:** mild cognitive impairment, cerebral small vessel disease, MRI, white matter hyperintensity, enlarged perivascular spaces, cerebral microbleed, cerebral atrophy

## Abstract

**Objectives:** Cerebral small vessel disease (CSVD) is the most common vascular cause of dementia, and mild cognitive impairment (MCI) is an intermediate state between dementia and normal cognitive aging. The present study investigated the main imaging features of CSVD on different MCI subtypes in memory clinics.

**Methods:** A total of 236 patients with MCI and 85 healthy controls were included. One hundred nine amnestic MCI-multiple domains (amMCI), 38 amnestic MCI-single domain (asMCI), 36 non-amnestic MCI-multiple domains (namMCI), and 53 non-amnestic MCI-single domain (nasMCI) patients were diagnosed. All participants were evaluated with the cognitive assessments and imaging features including white matter hyperintensity (WMH), enlarged perivascular spaces (EPVS), cerebral microbleeds (CMBs), and cerebral atrophy according to a standard procedure.

**Results:** The patients with amMCI, namMCI, and nasMCI had more high-grade basal ganglia EPVS compared with healthy controls, while the percentages of high-grade basal ganglia EPVS in the patients with amMCI were also more than those in patients with asMCI, namMCI, and nasMCI. There were more high-grade centrum semiovale EPVS in patients with amMCI in comparison with all other groups. The patients with amMCI and namMCI had more percentages of severe deep and periventricular WMH and deep CMBs compared with healthy controls. All MCI groups had higher scores of the medial temporal lobe atrophy than healthy controls, whereas the scores of the amMCI group were also higher than those of the namMCI and nasMCI groups.

**Conclusions:** There were varied neuroimaging features of CSVD including cerebral atrophy in different MCI groups, which meant that vascular mechanism contributed to the prodromal stage of dementia.

## Introduction

Dementia has become an important health problem among the aging population in China with 249.49 million people aged 60 years or older. Overall age-adjusted and sex-adjusted prevalence was estimated to be 6.0% for dementia, 3.9% for Alzheimer's disease (AD), and 1.6% for vascular dementia in people aged 60 years or older in China (1). Mild cognitive impairment (MCI) is considered as an intermediate state between dementia and normal cognitive aging with prevalence of 15.5% and could provide important information about the population at risk for developing dementia (1, 2). The concept of MCI was expanded to four subtypes, namely, amnestic MCI-single domain (asMCI), amnestic MCI-multiple domains (amMCI), non-amnestic MCI-single domain (nasMCI), and non-amnestic MCI-multiple domains (namMCI), which differ in etiology and outcome. Amnestic MCI (aMCI) is thought to have a high likelihood of progressing to AD, especially amMCI. Non-amnestic MCI (naMCI) is assumed to have a higher likelihood of progressing to a non-AD dementia (2, 3). Cerebral small vessel disease (CSVD) is a disorder of the brain's small perforating arterioles, capillaries, and probably venules that causes various lesions that are seen on pathological examination or brain imaging with magnetic resonance imaging (MRI) or computed tomography (CT). Typical CSVD includes white matter hyperintensities (WMH), enlarged perivascular spaces (EPVS), cerebral microbleeds (CMBs), and so on (4–6). Brain atrophy occurs with the usual aging process with heterogeneous pathological changes. Brain atrophy was thought to have a close relationship with neurodegenerative diseases, but many studies reported an association between atrophy and CSVD (4). Neurodegenerative diseases such as AD commonly coexist with cerebrovascular disease in older people, especially CSVD. CSVD could cause cognitive impairments and is a common cause of dementia, but the relationship has been questioned between CSVD and the various MCI types.

The purpose of the present study was to investigate the main imaging features of CSVD on different MCI subtypes.

## Materials and Methods

### Subjects

Three hundred twenty-one subjects were recruited from the memory clinics between 2015 and 2019. All participants underwent routine assessments, including standardized history taking, physical and neurological examinations, necessary laboratory tests, and an MRI scan. Of these participants, 236 of them were diagnosed with MCI and fulfilled the following criteria: (1) cognitive complaint, preferably corroborated by an informant; (2) objective cognitive impairment, quantified as a performance score more than 1.5 standard deviation (SD) below the appropriate mean on one cognitive test of any domain; (3) essentially intact activities of daily living (ADL); and (4) no dementia (2, 3). Subjects with a score < 1.5 SD from the mean on a learning measure, immediate or delayed recall, or recognition on the Rey Auditory Verbal Learning Test (RAVLT) (7) or immediate or delayed recall on the Rey–Osterrieth complex figure (ROCF) (7) were classified as having aMCI. Subjects with a score <1.5 SD from the mean on at least one test of attention, executive function, language facilities, and visuospatial capacity, but no memory impairment, were classified as having naMCI. Subjects with aMCI were subclassified as asMCI if they only had impairment in memory and as amMCI if they also had impairments in non-memory domains. Subjects with naMCI were subclassified as nasMCI if they only had impairment in one domain of non-memory domains and as namMCI if they also had impairments in two or above domains of non-memory domains. A total of 109 amMCI, 38 asMCI, 36 namMCI, and 53 nasMCI patients were included in the study.

Eighty-five subjects were considered as healthy controls. The inclusion criteria for the controls were as follows: (1) almost normal cognitive functions verified by informants; (2) the Mini Mental State Examination (MMSE) scores equal to or above 26 (8); (3) intact ADL; and (4) a Clinical Dementia Rating Scale (CDR) score of 0 (9). The exclusion criteria for the patients and controls were severe medical illness, neurological disorder, psychiatric disease, hearing or eyesight loss, and obvious abnormalities visible by cranial MRI. Participants who had been prescribed psychiatric drugs were also excluded.

The objectives of the research were explained to the participants and their families, and written informed consent was obtained for each participant. The research was approved by the Ethics Committee of the Beijing Tiantan Hospital.

### Clinical Evaluations

#### Cognitive Assessments

The cognitive assessments were administered by technicians according to a standard procedure and scored by a neuropsychologist. The time required for test administration was ~90 min.

The test battery included global cognitive screening, attention/processing speed, executive function, memory aptitude, language facilities, and visuospatial abilities. The MMSE and the Montreal Cognitive Assessment (MoCA) (Beijing version) (10) were used for global cognitive screening. The Digit Span Forward subset of the Wechsler Adult Intelligence Test-Revised Chinese version (WAIS-RC) (11), the Trail Making Test A (TMT-A) (7), the Stroop Color–Word Test (modified version) (SCWT) Part D (7), and the Digit Symbol subtest of the WAIS-RC (11) were used to assess processing speed/attention. Executive function was assessed using the Digit Span Backward subset of the WAIS-RC, the Chinese Version of Trail Making Test B (TMT-B) (12), and the SCWT Part C (7). The RAVLT including learning measure (the sum of trials 1–5), immediate and delayed recall, and recognition, and the ROCF including immediate and delayed recall were used to detect memory (7). The Semantic Category Verbal Fluency Test (animal) (7) and the Boston Naming Test (BNT) as modified by Cheung et al. (13) were employed to assess language ability. Visuospatial skills were verified by the copy part of the ROCF (7), the Block Design of the WAIS-RC (11), and the Clock Drawing Test (CDT) (14) scored by the Rouleau system. The raw scores were documented in all cognitive tests.

#### MRI

All subjects were scanned with a standardized scan protocol on 1.5- or 3.0-Tesla MRI scanners, including T1-weighted imaging, T2-weighted imaging, diffusion weighted imaging, fluid-attenuated inversion recovery sequence (FLAIR), and susceptibility-weighted imaging (SWI). All scans were visually rated by two trained neurologists who were blinded for the diagnosis.

EPVS were defined as fluid-filled spaces with a signal intensity similar to CSF on all sequences, which followed the course of penetrating vessels. They appeared linear, round, or ovoid, with a diameter generally smaller than 3 mm. EPVS were rated using a four-point visual rating scale on axial-T2-weighted images (1: <10 EPVS; 2: 11–20 EPVS; 3: 21–40 EPVS; and 4: >40 EPVS) for the basal ganglia (BG) and centrum semiovale (CSO) (15). The numbers of EPVS were counted in the slice with the highest number. The numbers refer to EPVS on one side of the brain, and the higher score was used if there was asymmetry between both hemispheres. The interrater reliability for the whole group was 0.753 and 0.807 for the scores of BG and CSO EPVS, respectively (*p* < 0.001).

WMH were hyperintense on T2-weighted sequences or FLAIR images. The degree of the severity was rated on FLAIR images according to the Fazekas score (range 0–3) (16). Periventricular (PV) and deep white matter hyperintensities (DWMH) were scored separately. The PV WMH score was 0 (absence), 1 (caps or pencil-thin lining), 2 (smooth halo), or 3 (irregular PV lesions extending into the deep white matter). The DWMH score was 0 (absence), 1 (punctate foci), 2 (beginning confluence of foci), or 3 (large confluent areas). The interrater reliability for the whole group was 0.771 and 0.816 for the scores of PV WMH and DWMH, respectively (*p* < 0.001).

CMBs were defined as small (generally 2–5 mm in diameter, but up to 10 mm) areas of signal void with associated blooming seen on SWI and were generally not seen on FLAIR, T1-weighted, or T2-weighted sequences. CMBs were classified manually as lobar CMBs (suggestive of cerebral amyloid angiopathy) and deep or infratentorial CMBs (suggestive of hypertensive arteriopathy) (17). The former included different cortical regions, and the latter included the basal ganglia, thalamus, internal capsule, external capsule, corpus callosum, deep and periventricular white matter, brainstem, and cerebellum. The interrater reliability for the whole group for the presence of CMBs was 0.882 (*p* < 0.001).

T1-weighted or FLAIR images were used to investigate regional brain atrophy by three visual rating scales: the medial temporal lobe atrophy (MTA) scale (18), the posterior atrophy (PA) scale (19), and the global cortical atrophy scale-frontal (GCA-F) subscale (20). The MTA was rated based on the five-point scale for the left or right side, respectively (0 = normal, 1 = widened choroid fissure, 2 = increase of widened fissure, widening temporal horn, opening of other sulci, 3 = pronounced volume loss of hippocampus, 4 = end stage atrophy). The PA was rated using the posterior cortical atrophy scale (range 0–3) with the average score of the left and right sides (0 = no atrophy, 1 = mild atrophy, opening of sulci, 2 = moderate atrophy, volume loss gyri, 3 = severe atrophy, knife blade). The GCA-F was assessed visually on the scale (range 0–3) (0 = no atrophy, 1 = mild atrophy, opening of sulci, 2 = moderate atrophy, volume loss gyri, 3 = severe atrophy, knife blade). The interrater reliability for the whole group was 0.885, 0.813, 0.900, and 0.846 for the scores of GCA-F, MTA of left or right side, and PA, respectively (*p* < 0.001).

### Statistical Analysis

Statistical analyses were performed using SPSS, version 17.0 (SPSS Inc., USA). Data are expressed as the mean ± SD unless otherwise specified. One-way analysis of variance (ANOVA) was applied for quantitative demographic variables among the control, amMCI, asMCI, nasMCI, and namMCI groups. We compared the results of the cognitive tests using analysis of covariance (ANCOVA) adjusted for age and education. Because multiple cognitive tests were administered, Bonferroni correction for multiple tests was also applied (*p* < 0.0025). The scores of brain atrophy were also assessed among all groups using ANOVA including the MTA, PA, and GCA-F.

The chi-square test was used to compare the differences between the qualitative variables among five groups, such as the sex ratio. The tests were also used to assess frequency distributions of neuroimaging variables (WMH, CMBs, and EPVS) in different MCI subgroups and healthy controls. All groups were dichotomized according to the score of PV or DMH (≧2 or <2), lobar or deep or infratentorial CMBs (absent or present), and the scores of EPVS for BG or CSO (≧2 or <2).

The Spearman correlation coefficient (r) was used to evaluate the correlations between imaging variables, including WMH, EPVS, MTA, PA, and GCA-F, and age, education, and scores of cognitive tests in all MCI groups. Binary logistic regression analysis was also used to assess the relationships between CMBs and age, education, and scores of cognitive tests and other imaging variables.

All statistical tests were two-tailed, and *p* < 0.05 was considered to indicate statistical significance.

## Results

### Comparison of Demographic and Cognitive Data Among Groups

The demographic and clinical data of the patients with amMCI, asMCI, namMCI, nasMCI, and the healthy controls are summarized in Table 1.

**Table 1:** Demographic, cognitive data of the patients with different MCI subtype and the healthy controls.

**Items**	**amMCI (*n* = 109)**	**asMCI(*n* = 38)**	**namMCI (*n* = 36)**	**nasMCI(*n* = 53)**	**Controls (*n* = 85)**	***P***	**Comparison among groups**
Age (years)	74.52 ± 8.25	74.66 ± 8.47	73.86 ± 10.30	71.30 ± 8.79	68.64 ± 8.62	<0.001	Controls < amMCI, asMCI, namMCI; nasMCI < amMCI
Gender (male %)	45.9	50.0	52.8	49.1	38.8	0.58	
Education (years)	13.08 ± 3.62	14.68 ± 2.37	13.17 ± 3.18	14.43 ± 2.92	14.41 ± 2.21	0.004	amMCI < controls, asMCI
MMSE	26.14 ± 2.16	27.16 ± 1.88	27.44 ± 2.24	27.70 ± 1.71	28.55 ± 1.35	<0.001	amMCI < controls; nasMCI
MoCA	21.23 ± 3.27	23.76 ± 2.55	22.59 ± 3.56	24.37 ± 2.61	25.80 ± 2.28	<0.001	amMCI, namMCI < controls; amMCI < asMCI, nasMCI
Digit Span Forward	7.37 ± 1.14	8.00 ± 0.66	6.89 ± 1.30	7.34 ± 1.47	8.20 ± 0.86	<0.001	namMCI, nasMCI < controls; namMCI < asMCI
TMT-A	86.35 ± 37.12	58.32 ± 18.83	83.17 ± 29.86	65.36 ± 24.74	49.19 ± 14.77	<0.001	Controls < amMCI, namMCI; asMCI < amMCI
SCWT Part D	20.23 ± 7.70	15.49 ± 4.14	19.69 ± 6.10	17.85 ± 5.61	14.05 ± 3.32	<0.001	Controls < amMCI, namMCI
Digit Symbol	26.06 ± 8.08	36.16 ± 9.76	27.33 ± 7.65	33.15 ± 8.72	39.41 ± 9.59	<0.001	amMCI, namMCI, nasMCI < controls; amMCI, namMCI < asMCI
Digit Span Backward	4.18 ± 1.14	4.92 ± 0.94	4.11 ± 0.92	4.66 ± 1.22	5.40 ± 1.30	<0.001	amMCI, namMCI < controls
TMT-B	233.07 ± 132.20	116.47 ± 42.89	205.31 ± 100.28	141.55 ± 62.28	93.25 ± 38.20	<0.001	Controls, asMCI < amMCI, namMCI; nasMCI < amMCI
SCWT Part C	40.86 ± 14.74	31.43 ± 6.27	37.57 ± 13.47	33.77 ± 9.82	28.39 ± 7.36	<0.001	Controls, asMCI < amMCI
Learning measure of RAVLT	27.64 ± 7.90	29.63 ± 8.27	39.64 ± 7.08	40.51 ± 7.80	42.73 ± 8.18	<0.001	amMCI, asMCI < controls, namMCI, nasMCI
Immediate recall of RAVLT	4.43 ± 2.65	5.18 ± 3.10	8.56 ± 2.10	8.53 ± 2.42	9.05 ± 2.27	<0.001	amMCI, asMCI < controls, namMCI, nasMCI
Delayed recall of RAVLT	3.03 ± 2.88	3.89 ± 2.66	7.31 ± 2.14	7.89 ± 2.69	8.20 ± 2.57	<0.001	amMCI, asMCI < controls, namMCI, nasMCI
Recognition of RAVLT	8.37 ± 3.80	8.97 ± 2.84	13.39 ± 1.50	13.06 ± 1.61	13.38 ± 1.40	<0.001	amMCI, asMCI < controls, namMCI, nasMCI
Immediate recall of ROCF	11.09 ± 7.09	14.03 ± 7.38	19.30 ± 4.22	21.14 ± 4.55	23.48 ± 5.16	<0.001	amMCI, asMCI < controls, namMCI, nasMCI
Delayed recall of ROCF	9.31 ± 7.40	13.33 ± 7.11	19.64 ± 3.63	20.34 ± 4.16	22.79 ± 5.01	<0.001	amMCI, asMCI < controls, namMCI, nasMCI
Verbal fluency	14.40 ± 3.85	17.95 ± 3.06	14.97 ± 4.50	17.79 ± 3.76	18.96 ± 3.42	<0.001	amMCI, namMCI < control; amMCI < asMCI, nasMCI
BNT	21.10 ± 3.62	24.47 ± 2.40	20.94 ± 3.55	24.02 ± 3.82	25.08 ± 2.39	<0.001	amMCI, namMCI < controls, asMCI, nasMCI
Copy part of ROCF	32.63 ± 3.81	34.33 ± 1.89	33.78 ± 2.39	33.85 ± 3.20	34.64 ± 1.66	<0.001	amMCI < controls
Block design	24.39 ± 6.67	29.42 ± 6.15	26.19 ± 7.00	29.49 ± 6.45	33.14 ± 6.74	<0.001	amMCI, namMCI, < controls
CDT	8.19 ± 2.01	9.47 ± 0.80	7.86 ± 2.49	9.00 ± 1.44	9.61 ± 0.68	<0.001	amMCI, namMCI < control, asMCI

The healthy controls were younger than patients with amMCI, asMCI, and namMCI, whereas amMCI patients were slightly older than nasMCI patients. There were no differences in age among other groups. Patients with amMCI had lower education levels than patients with asMCI and healthy controls, but there were no differences in education among other groups. There were no sex differences among different groups.

After adjusting age and education, patients with amMCI performed worse than patients with nasMCI and healthy controls on the MMSE (Bonferroni correction was applied and level of significance set at 0.0025). The scores of the MoCA in patients with amMCI and namMCI were lower than those of healthy controls, whereas patients with amMCI also had worse scores than patients with asMCI and nasMCI.

On the attention/processing speed domain, the Digit Span Forward scores in patients with namMCI and nasMCI were lower than those of healthy controls, while patients with namMCI also performed worse than patients with asMCI. Patients with amMCI and namMCI performed worse on the TMT-A and the SCWT Part D than healthy controls, whereas patients with amMCI spent a longer time on the TMT-A compared with patients with asMCI. Patients with amMCI, namMCI, and nasMCI had lower scores of the Digit Symbol than healthy controls, while patients with amMCI and namMCI did worse than patients with asMCI.

On the domain of executive function, the scores of patients with amMCI and namMCI were lower on the Digit Span Backward than those of healthy controls. Patients with amMCI and namMCI performed worse on the TMT-B than healthy controls and patients with asMCI, while the amMCI group also performed worse than the nasMCI group. As to SCWT Part C, healthy controls and patients with asMCI spent a shorter time than did patients with amMCI.

On the domain of memory, patients with amMCI and asMCI had lower scores than patients with namMCI and nasMCI and healthy controls on the learning measure, immediate and delayed recall, and recognition of the RAVLT. The scores of patients with amMCI and asMCI were also lower than those of patients with namMCI and nasMCI and healthy scores on immediate and delayed recall of the ROCF.

5On the domain of language facilities, patients with amMCI and namMCI had lower scores than patients with asMCI and nasMCI and healthy controls on the BNT. Patients with amMCI and namMCI performed worse than healthy controls on the verbal fluency test, while the scores of patients with amMCI were lower than those of patients with asMCI, nasMCI.

On the domain of visuospatial abilities, patients with amMCI and namMCI had lower scores on the Block Design than healthy controls. Patients with amMCI and namMCI performed worse than patients with asMCI and healthy controls on the CDT. As to the copy part of the ROCF, the scores of patients with amMCI were lower than those of healthy controls.

### Comparison of Imaging Data of CSVD Among Groups

We compared the percentages of participants with higher scores of PV WMH or DWMH, lobar or deep or infratentorial CMBs, higher scores of EPVS for BG or CSO, and the degree of brain atrophy represented as scores of MTA, PA, and GCA-F among groups.

**Figure 1:**
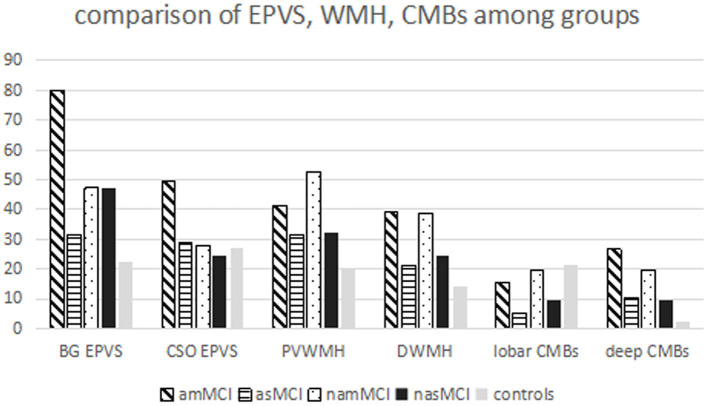
Comparison of EPVS, WMH, and CMBs among all groups.

The patients with amMCI, namMCI, and nasMCI had more high-grade BG EPVS compared with healthy controls, while the percentages of high-grade BG EPVS in the patients with amMCI were also more than those in patients with asMCI, namMCI, and nasMCI. There were more high-grade CSO EPVS in patients with amMCI in comparison with all other groups (Table 2, Figure 1).

**Table 2:** Imaging data of the patients with different MCI subtypes and the healthy controls.

**Items**	**amMCI(*n* = 109)**	**asMCI(*n* = 38)**	**namMCI(*n* = 36)**	**nasMCI(*n* = 53)**	**Controls(*n* = 85)**	***P***	**Comparison among groups**
BG EPVS (%)	79.8	31.6	47.2	47.2	22.4	<0.001	Controls < amMCI, namMCI, nasMCI; asMCI, namMCI, nasMCI < amMCI
CSO EPVS (%)	49.5	28.9	27.8	24.5	27.1	0.002	Controls, asMCI, namMCI, nasMCI < amMCI
PVWMH (%)	41.3	31.6	52.8	32.1	20.0	0.003	Controls < amMCI, namMCI
DWMH (%)	39.4	21.1	38.9	24.5	14.1	0.001	Controls < amMCI, namMCI; asMCI < amMCI
Lobar CMBs (%)	15.6	5.3	19.4	9.4	21.2	0.129	
Deep or infratentorial CMBs (%)	26.6	10.5	19.4	9.4	2.4	<0.001	Controls < amMCI, namMCI; asMCI, nasMCI < amMCI
GCA-F	1.35 ± 0.53	1.36 ± 0.55	1.31 ± 0.47	1.18 ± 0.39	1.05 ± 0.45	0.002	Control < amMCI
MTA	3.53 ± 1.36	3.36 ± 1.43	2.88 ± 1.37	3.00 ± 1.16	2.13 ± 1.34	<0.001	Controls < amMCI, asMCI, namMCI, nasMCI; namMCI, nasMCI < amMCI
Left MTA	1.73 ± 0.74	1.67 ± 0.82	1.46 ± 0.71	1.45 ± 0.60	1.00 ± 0.69	<0.001	Controls < amMCI, asMCI, nasMCI
Right MTA	1.80 ± 0.69	1.70 ± 0.68	1.42 ± 0.70	1.55 ± 0.60	1.13 ± 0.70	<0.001	Controls < amMCI, asMCI, namMCI, nasMCI; namMCI < amMCI
PA	1.45 ± 0.57	1.48 ± 0.67	1.58 ± 0.58	1.38 ± 0.59	1.22 ± 0.60	<0.001	Controls < amMCI, asMCI, namMCI

The patients with amMCI and namMCI had more severe DWMH compared with healthy controls, while patients with amMCI also had higher percentages of severe DWMH than patients with asMCI. There were marginal differences between amMCI and nasMCI (*p* = 0.061) and asMCI and namMCI (*p* = 0.093). Patients of amMCI and namMCI had more severe PVWMH than healthy controls, whereas the namMCI group also had higher percentages of severe PVWMH compared with the asMCI and nasMCI groups, although the differences were marginal (*p* = 0.065, 0.051) (Table 2, Figure 1).

Patients with MCI had 19.1% (45/236) deep or infratentorial and 13.1% (31/236) lobe CMBs. Patients with amMCI and namMCI had more deep or infratentorial CMBs than healthy controls, whereas there were also more deep or infratentorial CMBs in patients with amMCI compared with patients with asMCI and nasMCI. The differences between asMCI and nasMCI patients and healthy controls were marginal (*p* = 0.052, 0.065). As to lobar CMBs, there were no differences among groups (Table 2, Figure 1).

**Figure 2:**
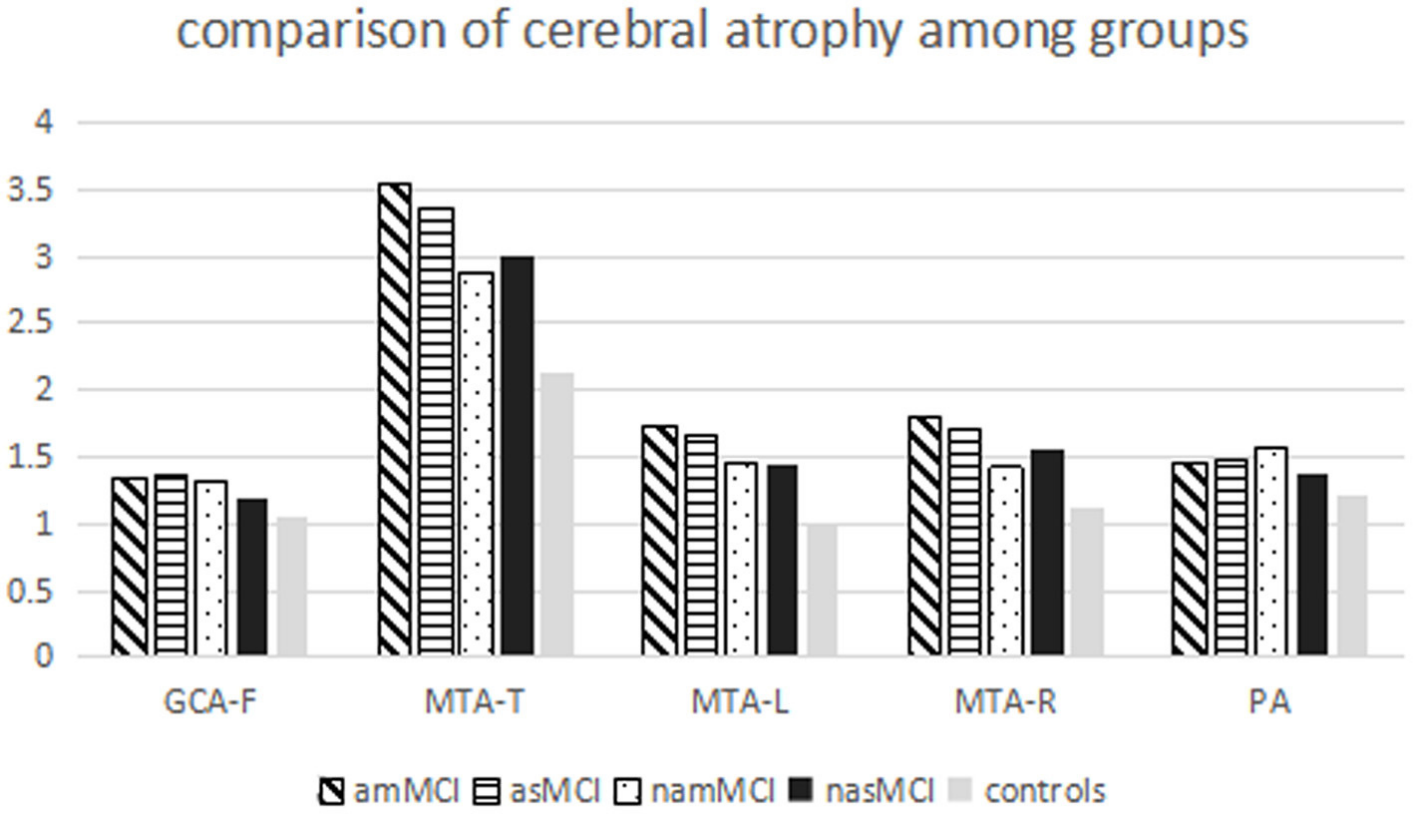
Comparison of cerebral atrophy among all groups.

All MCI groups had higher total scores of the MTA than healthy controls, whereas the scores of the amMCI group were also higher than those of the namMCI and nasMCI groups. Patients with amMCI, asMCI, and nasMCI had higher scores of left and right MTA than healthy controls, while there were higher scores of right MTA on patients with amMCI compared with patients with namMCI. Patients with amMCI had higher scores of GCA-F than healthy controls. There were high scores of PA on patients with amMCI, asMCI, and namMCI compared with healthy controls (Table 2, Figure 2).

### Correlations Between Variables of CSVD and Cognitive Tests

In MCI groups, BG EPVS had positive correlations with white matter hyperintensities and frontal and posterior atrophy in addition to CSO EPVS, while there were correlations between BG EPVS and the MoCA, and tests of cognitive domains including attention/processing speed, executive function, verbal memory, language facilities, and visuospatial abilities. CSO EPVS had positive correlations with white matter hyperintensities, while there were only correlations between CSO EPVS and learning measures of RAVLT, the verbal fluency, and the SCWT Part D.

PVWMH had positive correlations with medial temporal lobe atrophy in addition to DWMH, whereas DWMH had positive correlations with medial temporal lobe and posterior atrophy. There were correlations between WMH and age and tests involving attention/processing speed and executive function.

Frontal atrophy had positive correlations with medial temporal lobe and posterior atrophy, whereas there was a positive relationship between medial temporal lobe atrophy and posterior atrophy. Frontal atrophy had correlations with age, education, the MMSE, and tests including attention/processing speed, executive function, and verbal and visuospatial memory. Medial temporal lobe atrophy also had correlations with age, education, the MMSE, and tests including attention/processing speed, executive function, and verbal and visuospatial memory. Posterior atrophy only had correlations with age, education, and tests including attention/processing speed, executive function, and learning measures of RAVLT (Table 3).

**Table 3:** Correlation between imaging variables and age, education, and scores of cognitive tests in MCI groups.

**Items**	**BG EPVS**	**CSO EPVS**	**PV WMH**	**DWMH**	**GCA-F**	**MTA**	**PA**
Age	0.122	0.109	0.276^a^	0.211^a^	0.465^a^	0.455^a^	0.452^a^
Education	−0.092	0.015	0.131	0.083	0.228^a^	0.234^a^	0.201^a^
BG EPVS		0.362^a^	0.232^a^	0.260^a^	0.224^a^	0.115	0.212^a^
CSO EPVS	0.362^a^		0.159^a^	0.239^a^	−0.006	0.005	0.098
PV WMH	0.232^a^	0.159^a^		0.783^a^	0.085	0.194^a^	0.117
DWMH	0.260^a^	0.239^a^	0.783^a^		0.046	0.178^a^	0.147^a^
GCA-F	0.224^a^	−0.006	0.085	0.046		0.312^a^	0.661^a^
MTA	0.115	0.005	0.194^a^	0.178^a^	0.312^a^		0.232^a^
PA	0.212^a^	0.098	0.117	0.147^a^	0.661^a^	0.232^a^	
MMSE	−0.024	−0.033	−0.097	−0.091	−0.152^a^	−0.148^a^	−0.121
MoCA	−0.179^a^	−0.037	−0.073	−0.162^a^	0.123	−0.065	−0.066
Digit span forward	0.022	0.010	−0.050	−0.018	0.043	−0.136	0.026
TMT-A	0.181^a^	0.083	0.203^a^	0.175^a^	0.142	0.207^a^	0.104
SCWT part D	0.230^a^	0.183^a^	0.116	0.121	0.113	0.101	0.148^a^
Digit symbol	−0.208^a^	−0.064	−0.186^a^	−0.194^a^	−0.173^a^	−0.199^a^	−0.148^a^
Digit span backward	−0.017	−0.004	−0.114	−0.082	0.011	0.012	−0.003
TMT-B	0.219^a^	0.045	0.177^a^	0.243^a^	0.207^a^	0.160^a^	0.184^a^
SCWT part C	0.107	0.044	0.122	0.100	0.015	0.149^a^	0.054
Learning measure of RAVLT	−0.199^a^	−0.222^a^	−0.073	−0.092	−0.236^a^	−0.252^a^	−0.174^a^
Immediate recall of RAVLT	−0.145	−0.119	−0.015	−0.044	−0.164^a^	−0.185^b^	−0.111
Delayed recall of RAVLT	−0.190^a^	−0.124	−0.092	−0.102	−0.157^a^	−0.219^a^	−0.091
Recognition of RAVLT	−0.125	−0.138	0.063	0.004	−0.188^a^	−0.123	−0.084
Immediate recall of ROCF	−0.101	−0.150	−0.005	−0.091	−0.155^a^	−0.181^a^	−0.026
Delayed recall of ROCF	−0.106	−0.136	0.036	−0.044	−0.159^a^	−0.202^a^	−0.027
Verbal fluency	−0.148	−0.155^a^	−0.045	−0.155^a^	−0.010	−0.047	−0.029
BNT	−0.263^a^	−0.095	−0.069	−0.037	−0.047	0.054	−0.076
Copy part of ROCF	−0.163^a^	−0.077	0.035	−0.051	−0.091	−0.008	−0.105
Block design	−0.153	0.001	−0.075	−0.056	−0.046	−0.050	0.026
CDT	−0.068	0.158^a^	−0.070	−0.081	0.036	−0.031	0.121

a *p < 0.05*.

Binary logistic regression analysis showed that only age contributed to lobar CMBs (OR = 1.160, 95 CI 1.035–1.301, *p* = 0.011). There was no relationship between deep or infratentorial CMBs and any other imaging and cognitive indicators.

## Discussion

The present study investigated cognitive and imaging features of CSVD in MCI. There were obvious cognitive impairments in different MCI groups, especially amMCI. Different imaging changes of CSVD were also shown in various MCI patients.

Patients with aMCI had obvious memory impairments than naMCI and controls on all memory tests. There were heterogeneous results on different tests of other domains. The multiple-domain MCI group performed worse on executive function and visuospatial abilities compared with controls. Patients with amMCI and naMCI had worse scores than controls on the attention domain, while patients with multiple-domain MCI performed worse on the language domain compared with single-domain MCI and controls.

The patients with amMCI had more high-grade BG PVS followed by patients with namMCI and nasMCI compared with healthy controls, while there were more high-grade CSO PVS in patients with amMCI in comparison with all other groups. EPVS had obvious correlations with PV WMH and DWMH, whereas BG EPVS also had relationships with frontal and parietal atrophy. There was a correlation between BG EPVS and every domain of cognition tests, but COS EPVS had only a relationship with attention, language, and learning parts of the verbal memory test.

Perivascular spaces are extensions of the extracerebral fluid space around arteries, arterioles, veins, and venules, which are the main drainage conduits and form part of the glymphatic system (4). EPVS could involve impairment of cerebrovascular reactivity, blood–brain barrier dysfunction, perivascular inflammation, and abnormal clearance of waste proteins from the interstitial fluid space, ultimately leading to accumulation of toxins, hypoxia, and tissue damage (21). All pathophysiological processes could increase WMH, affect clearance of β amyloid, and cause its accumulation in the brain, which increase the risk of cognitive decline and dementia and play a potential key role on the pathogenesis of AD. Previous studies found that EPVS was associated with WMH but not atrophy (4, 22). We demonstrated it in patients with MCI, but the relationship between BG EPVS and frontal and parietal atrophy was also reported. We found that EPVS of various regions had different effects on cognition and MCI. Patients with amMCI had more high-grade BG and CSO EPVS, whereas there was more high-grade BG EPVS in only naMCI patients. A Spanish study reported that BG EPVS not CSO could predict MCI in hypertensive individuals although affected by other markers of CSVD (23). The scores of BG EPVS were higher in vascular dementia than those of AD, and there were no differences on CSO EPVS scores (24). Another study showed that a high degree of white matter EPVS is associated with the number of lobar MBs, while a high degree of BG EPVS is associated with the presence of hypertension. The results meant that white matter EPVS had a relationship with cerebral amyloid angiopathy (CAA) (25). CSO EPVS might indicate the presence of CAA or a mixed hypertensive/CAA. However, we found no correlation between EPVS and CMBs, which may contribute the method assessing CMBs. Participants with severe EPVS in both regions or in the CSO alone had greater decline in global cognition, and the presence of severe EPVS in both regions was an independent predictor of dementia. The study did not find the association between cognitive domain and EPVS (26). Our study found that BG EPVS had a relationship with more extensive cognitive domain compared with CSO EPVS.

The patients with amMCI and namMCI had more percentages of severe DWMH and PVWMH compared with healthy controls. There were more severe DWMH in patients with amMCI compared with asMCI. There were marginal differences between amMCI and nasMCI and namMCI and asMCI in DWMH, and namMCI and asMCI or nasMCI in PVWMH. WMH had a correlation with attention, executive function, and medial temporal lobe atrophy.

The pathogenesis of WHM is not well-understood and could be multifactorial, although strongly associated with cerebrovascular disease and vascular risk factors (4). In the baseline cognitively normal individuals, greater WMH were associated with accelerated multiple-domain cognitive, neuropsychiatric, and functional decline independent of traditional risk factors. WMH also contributed to the development of MCI (27, 28). There was no statistical difference within various MCI types regarding DWMH, while PVWMH scores were significantly higher in patients with naMCI than those in patients with aMCI (29). The aMCI group showed elevated temporal and occipital WMH volume relative to the control group whereas the naMCI group showed elevated WMH volume across frontal, parietal, temporal, and occipital regions, suggesting more widespread WMH accumulation. In addition, the naMCI participants showed greater occipital WMH relative to the aMCI (30). Our study demonstrated that the percentages of high-grade PVWMH and DWMH were higher in multiple-domain MCI compared with single-domain MCI or healthy controls.

The meta-analysis showed that the association between WMH and overall cognition was significantly stronger for MCI than for AD (31). For both groups, the largest effect sizes were found in attention and executive functions and processing speed. Interestingly, there was also a significant association with the memory domain which is more closely related to AD. The study also suggested that PV WMH were more strongly related to cognition than DWMH. DWMH related to ischemic risk factors may predominantly disrupt the short association fibers, which linked to adjacent gyri. PVWMH linked to atrophic processes was likely to affect the long association fibers that connected the more distant cortical areas. The study involving executive functions revealed the association between PVWMH and working memory, DWMH and inhibition performance, and MTA and flexibility performance (32). The relationship between WMH and attention and executive function was also demonstrated in our study.

The MRI study showed that hippocampal subfield atrophy worsened with increasing CSVD severity, mainly WMH. Greater atrophy was seen with moderate to severe CSVD compared to mild CSVD in the subfields including the subiculum, CA1, CA4, molecular layer, and dentate gyrus. Atrophy in the subfields was significantly associated with poor episodic memory and frontal executive function (33). The present study also certified that WMH had an obvious correlation with MTA.

Patients with amMCI and namMCI had more deep or infratentorial CMBs than healthy controls, whereas there were also more deep or infratentorial CMBs in patients with amMCI compared with patients with asMCI and nasMCI. The differences between asMCI and nasMCI patients and healthy controls were marginal. As to lobar CMBs, there were no differences among groups.

CMBs may represent hemosiderin-laden macrophages in perivascular tissue, secondary to vascular leakage of blood cells. Deep or infratentorial CMBs were hypothesized to be associated with hypertensive microangiopathy, while lobar CMBs may be due to CAA (4). The mechanisms of the association between CMBs and cognitive dysfunction were not well-understood.

Patients with AD and progress subtype of MCI had significantly more new CMB than controls and patients with a stable subtype of MCI during the follow-up (34). Total number of CMBs and of those in deep and lobar regions were associated with attention and executive function and fluency domains (35). The presence of any CMBs, including lobar and deep or infratentorial CMBs, was related to MCI after adjusting for confounders. Furthermore, the presence of multiple microbleeds is associated with lower MoCA total scores and with worse performance on specific domains of cognitive tests, such as global cognitive function, information processing speed, and motor speed (36). Our patients with MCI had 19.1% deep or infratentorial and 13.1% lobe CMBs, which was similar to a previous study (17). There were more deep or infratentorial CMBs in various MCI groups, while those in the amMCI group was also more obvious than the single-domain MCI group. There was no difference in lobe CMBs, which may contribute to high percentages of lobe CMBs in healthy controls selected from memory clinics. We did not find the association between CMBs and cognition, which could be caused by the method evaluating CMBs only absent or present.

Although brain atrophy was attributed to neurodegenerative diseases, many imaging studies reported an association between the presence and severity of SVD and brain atrophy, even including hippocampal atrophy. Moreover, atrophy was an important measure in imaging studies that were done to assess the burden of vascular damage in the brain and cognition (4). Brain atrophy was present in all MCI types and was greater in multiple-domain types particularly in the naMCI (29, 37). The present study found that all MCI groups had obvious atrophy of the medial temporal lobe compared with healthy controls, whereas the difference was also obvious in the amMCI group than the naMCI group. Patients with amMCI had obvious frontal atrophy, while there was a prominent parietal atrophy in patients with aMCI and namMCI in comparison with healthy controls. Episodic memory is often considered as a marker of early stage in patients with AD. Scores of episodic memory tests were reported to correlate with hippocampal volumes in MCI, which also were associated with executive function (33, 38). Our study showed that frontal and medial temporal lobe atrophy had an obvious correlation with attention, executive function, and learning and memory, whereas there was a relationship between parietal atrophy and attention, executive function and learning part of verbal memory.

The present study has some limitations. First, the participants were selected from our memory clinics, not from the community, which may lead to bias. Second, more patients with amMCI were selected compared with asMCI and namMCI. Thirdly, the healthy controls were younger, and patients with amMCI had lower education levels, but we analyzed the results after adjusting the age and education.

In conclusion, patients with different MCI types had obvious cognitive impairments, especially amMCI. The imaging of CSVD with different frequencies, such as EPVS, WMH, CMBs, and brain atrophy, was found in various MCI groups. There was a relationship between varied neuroimaging features of CSVD and cognitive impairment. All of those meant that the vascular mechanism contributed to the prodromal stage of dementia.

## Data Availability Statement

The raw data supporting the conclusions of this article will be made available by the authors, without undue reservation.

## Ethics Statement

The studies involving human participants were reviewed and approved by the Ethics Committee of the Beijing Tiantan Hospital. The patients/participants provided their written informed consent to participate in this study.

## Author Contributions

XL, XT, and JJ contributed to the conception and design of the study. XL analyzed and interpreted the data. XL and XT revised the manuscript. MS, YJ, SJ, ZZ, ZH, and XZ contributed to the participants' enrollment and the clinical assessments. MS wrote the first draft of the manuscript. All the authors contributed to the article and approved the submitted version.

## Conflict of Interest

The authors declare that the research was conducted in the absence of any commercial or financial relationships that could be construed as a potential conflict of interest. The handling editor declared a shared affiliation with several of the authors XL and ZH at time of review.

## Abbreviations

AD: Alzheimer's disease
ADL: activities of daily living
aMCI: amnestic mild cognitive impairment
amMCI: amnestic mild cognitive impairment-multiple domains
asMCI: amnestic mild cognitive impairment-single domain
BG: basal ganglia
BNT: Boston Naming Test
CDR: Clinical Dementia Rating Scale
CDT: Clock Drawing Test
CMBs: cerebral microbleeds
CSO: centrum semiovale
CSVD: cerebral small vessel disease
CT: Computed Tomography
DWMH: deep white matter hyperintensities
EPVS: enlarged perivascular spaces
FLAIR: fluid attenuated inversion recovery sequence
GCA-F: global cortical atrophy scale-frontal
MMSE: Mini Mental State Examination
MoCA: Montreal Cognitive Assessment
MRI: Magnetic Resonance Imaging
MTA: medial temporal lobe atrophy
naMCI: non-amnestic mild cognitive impairment
namMCI: non-amnestic mild cognitive impairment-multiple domains
nasMCI: non-amnestic mild cognitive impairment-single domain
PA: posterior atrophy
PV WMH: periventricular white matter hyperintensities
RAVLT: Rey Auditory Verbal Learning Test
ROCF: Rey-Osterrieth complex figure
SCWT: Stroop Color Word Test
SWI: susceptibility-weighted imaging
TMT-A: Trail Making Test A
TMT-B: Trail Making Test B
WMH: white matter hyperintensities.
